# Editorial: Telemedicine in cardiology

**DOI:** 10.3389/fcvm.2025.1665285

**Published:** 2025-08-04

**Authors:** Han Feng, Mario Mekhael, Duo Yu, Chanho Lim, Hongyu Miao, Nassir Marrouche

**Affiliations:** ^1^ Tulane Research Innovation for Arrhythmia Discovery (TRIAD), Tulane University School of Medicine, New Orleans, LA, United States; ^2^ Division of Biostatistics, Data Science Institute, Medical College of Wisconsin, Milwaukee, WI, United States; ^3^ Florida State University College of Nursing, Tallahassee, FL, United States

**Keywords:** telemedicine, AI, cardiovascular diseases, cardiology, digital health, screening and monitoring, cardiological procedures

**Editorial on the Research Topic**
Telemedicine in cardiology

Cardiology is gradually entering a phase of digital transformation, where clinical care and research are increasingly informed by remote technologies and algorithmic tools. While the integration of telemedicine into cardiovascular practice remains suboptimal, evidence continues to accumulate in support of its feasibility and clinical value. Recent literature emphasizes that meaningful digital integration in cardiovascular care is most effective when patient-centered (1–3). Furthermore, tools can be introduced at different stages of healthcare, whether prior to an intervention as a screening or diagnostic tool, or after the fact as a monitoring tool. The studies presented in this editorial reflect such efforts: patient-focused and aligned with the realities of current cardiovascular practice (Figure 1).

**Figure 1:**
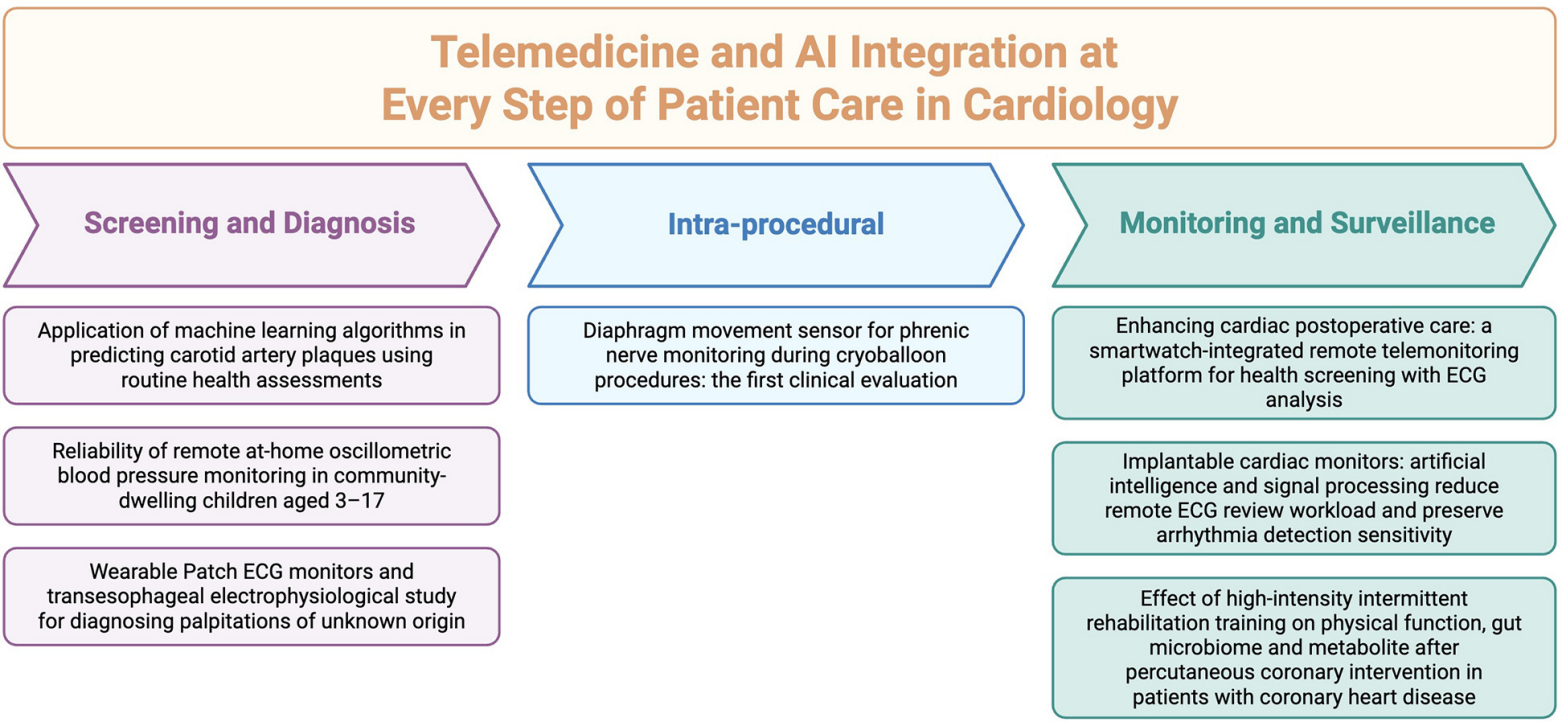
Central illustration of articles published under the theme “telemedicine in cardiology”, categorized into three domains: “screening and risk prediction”, “intra-procedure”, and “monitoring and rehabilitation”.

## Screening and risk prediction

Artificial intelligence (AI) may be particularly helpful in early prevention and screening stages. A large-scale machine learning study (Wei et al.) utilized algorithms such as LightGBM to analyze routine clinical and laboratory data, predicting carotid artery plaques with an AUC of 85.4%. This model eliminates the need for advanced imaging, thereby broadening access to early atherosclerosis risk stratification, particularly for underserved populations.

Access and scalability are central to the promise of telemedicine. Especially in prevention, where identifying patients at risk for a certain disease is crucial for effective screening strategies. A study on at-home oscillometric blood pressure monitoring in children aged 3–17 (Ho et al.) showed that caregivers could reliably assess blood pressure, particularly in normotensive children. This supports the idea that early cardiovascular risk screening can begin at home, potentially mitigating disease progression before it reaches the clinic. Also, this can further identify subjects that may need further workup and management.

Moreover, combining different tools can increase screening and diagnostic yield. For example, in rhythm diagnostics, combining wearable patch ECGs with transesophageal electrophysiology (TEPS) created a hybrid approach to evaluating palpitations of unknown origin (Yang et al.). In patients with negative TEPS, prolonged patch monitoring identified previously undetected arrhythmias. This complementary use of non-invasive and invasive technologies demonstrates how telecardiology can extend and personalize preoperative arrhythmia workups to improve patient care.

## Monitoring and rehabilitation

Wearables and implantable devices are rapidly becoming the backbone of outpatient cardiac monitoring. In a cohort of 108 cardiac surgery patients, a smartwatch-integrated platform tracked ECG, heart rate, and blood pressure from home (Monteiro et al.). It successfully detected asymptomatic AV block and other arrhythmias with strong concordance to in-clinic assessments. These findings highlight how consumer-grade devices are expanding into clinical territory, providing scalable tools for improving outcomes.

Similarly, implantable cardiac monitors augmented by the SmartECG algorithm addressed a longstanding challenge in telemonitoring: alert fatigue (Bisignani et al.). The algorithm filtered nearly 43% of false detections while maintaining a low 2.6% sensitivity loss, reducing clinician review time by over 40%. This balance of precision and efficiency illustrates how AI can support sustainable, long-term arrhythmia surveillance.

Likewise, the study of high-intensity interval training (HIIT) following PCI (Jiang et al.) provides a framework for home-based rehabilitation. HIIT led to notable improvements in VO2 peak and 6-minute walk test performance, especially in patients with prior myocardial infarction. These physiologic gains were paralleled by shifts in gut microbiome and metabolomics, pointing toward a future of biologically informed, remotely delivered exercise interventions.

## Procedural integration and intraoperative monitoring

Even within procedural cardiology, sensor-driven monitoring is beginning to influence care. A first clinical evaluation of a diaphragm movement sensor integrated into cryoballoon systems (Schemoul et al.) aimed to detect right phrenic nerve stress. While its diagnostic performance was limited, the concept reflects a growing emphasis on real-time, automated support tools that may one day enhance safety and standardization in electrophysiologic procedures.

## Conclusion: an AI-driven future at every step of patient care

This Research Topic offers a timely perspective on the evolving role of telemedicine in cardiovascular care. While still in its early stages, the integration of remote monitoring, wearable technologies, and AI is gradually reshaping both clinical practice and research (4). The studies highlighted here demonstrate practical, data-driven contributions from preoperative risk assessment to postoperative rehabilitation and procedural support. Rather than claiming revolution, this collection reflects steady progress: incremental yet meaningful steps toward distributed, accessible, and intelligent cardiovascular care. Importantly, these efforts are rooted in real-world contexts and designed to complement existing workflows and act as supportive tools. Admittedly, telemedicine is not intended to replace in-person interactions between patients and clinicians, but rather to extend the reach of care and ensure broader populations can benefit from available medical resources (5). Sustainable innovation in cardiology has been shown to depend on the translation of digital health technologies into clinically actionable tools that support both clinicians and patients (6). This Research Topic illustrates how such translation is beginning to take shape at every step of patient care.
